# Supplementary material for: Development of Solid Lipid Nanoparticles by Cold Dilution of Microemulsions: Curcumin Loading, Preliminary In Vitro Studies, and Biodistribution
Source: Nanomaterials (Basel). 2019 Feb 8;9(2):230. doi: 10.3390/nano9020230 (PMC6410061; doi:10.3390/nano9020230)
Supplement: Supplementary file 1 [file nanomaterials-09-00230-s001.pdf]

Supplementary

Development of Solid Lipid Nanoparticles by Cold Dilution of Microemulsions: Curcumin Loading, Preliminary In Vitro Studies, and Biodistribution

Daniela Chirio [1], Elena Peira [1,*], Chiara Dianzani [1], Elisabetta Muntoni [1], Casimiro Luca Gigliotti [2], Benedetta Ferrara [1], Simona Sapino [1], Giulia Chindamo [1] and Marina Gallarate [1]

[1] Dipartimento di Scienza e Tecnologia del Farmaco, Università degli Studi di Torino, 10125 Torino, Italy; daniela.chirio@unito.it (D.C.); chiara.dianzani@unito.it (C.D.); elisabetta.muntoni@unito.it (E.M.); benedetta.ferrara@unito.it (B.F.); simona.sapino@unito.it (S.S.); giulia.chindamo@edu.unito.it (G.C.); marina.gallarate@unito.it (M.G.)

[2] Interdisciplinary Research Center of Autoimmune Diseases, Department of Health Sciences, "A. Avogadro" University of Eastern Piedmont, 28100 Novara, Italy; luca.gigliotti@med.unipmn.it (C.L.G.)

* Corresponding Author: elena.peira@unito.it; Tel.: +39-011-670-7143

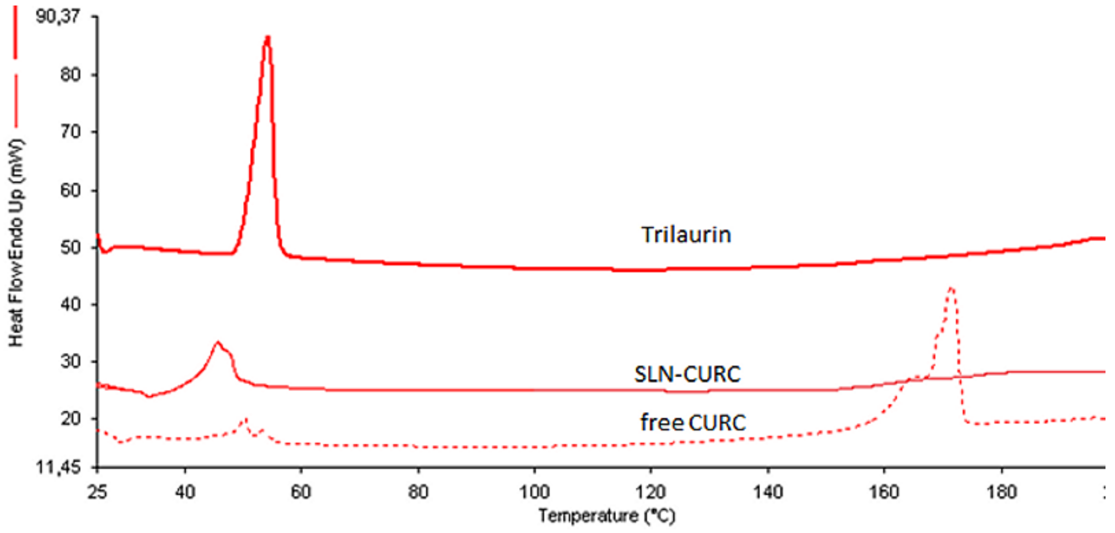

Figure 1. DSC thermograms of TL, CURC bulk material, and SLN-CURC after production and GF.
